# Supplementary material for: Tracking attosecond electronic coherences using phase-manipulated extreme ultraviolet pulses
Source: Nat Commun. 2020 Feb 14;11:883. doi: 10.1038/s41467-020-14721-2 (PMC7021897; doi:10.1038/s41467-020-14721-2)
Supplement: Supplementary file 1 — Supplementary Information [file 41467_2020_14721_MOESM1_ESM.pdf]

Supplementary information

Tracking Attosecond Electronic Coherences Using Phase-Manipulated Extreme Ultraviolet Pulses

A. Wituschek et. al

Supplementary Note 1: Phase retrieval using heterodyned detection:

The full characterization of an electron WP in amplitude and phase is a non-trivial problem and a major goal in attosecond metrology. The issue of dissecting the amplitude $A(t)$ and phase function $\phi(t)$ of an arbitrary signal $S(t) = A(t)e^{i\phi(t)}$ is well-known from e.g. optical pulse characterization, where many methods have been developed to solve this problem. However, it is difficult to apply these methods in the XUV spectral range, which makes the phase retrieval of WPs excited/probed in the XUV domain generally a challenging task.

We solve this problem by introducing heterodyned detection with a known reference waveform, a common method in signal processing. Instead of using optical heterodyning, which is challenging at XUV wavelengths, the imprinted phase modulation effectively shifts the signal down to the low kHz-frequency regime where standard lock-in electronics can be used for heterodyned detection with a known electronic waveform. The quadrature demodulation with the known reference then yields in-phase and in quadrature signal components from which amplitude and phase are readily reconstructed (see also Methods section).

We note that our approach for retrieving amplitude and phase of a WP is universal and does not require energy-resolved detection. This permits its implementation with arbitrary detection types like ion time-of-flight detection, velocity map imaging or reaction microscopes.

Supplementary Note 2: Contribution of the Fano profile to the ion/electron count rate:

In quantum interference experiments, typically the pathway interference between the pump excitation and the delayed probe excitation is probed. For the presented study of a Fano resonance, the situation is slightly more complex, as each pulse can excite two coherent pathways leading to the same final state (labeled $i = 1, 2$ in supplementary Fig. 2a). For pathway amplitudes A_i the ion/electron signal is accordingly

$$S \propto |A_1(t) + A_2(t) + A_1(t + \tau) + A_2(t + \tau)|^2 \, . \quad (1)$$

Here, the interference term of pathway i excited by the pump with pathway j excited by the

delayed probe ($i, j = 1,2, i \neq j$) contributes the phase difference between A_1 and A_2 to the signal which is hence encoded in the measured ion/electron count rates.

For a quantitative derivation of the signal we apply time-dependent perturbation theory and adapt the calculations from supplementary Ref. 1 to a one-dimensional quantum interference experiment. The experimental observable is the ion/electron yield which is proportional to the population probability of the quasi-bound eigenstates $|k\rangle$ of the diagonalized Hamiltonian (supplementary Fig. 2b). Expanding the signal to second-order in the optical field and assuming delta-like excitation pulses yields:

$$S(\tau) \propto \int \mathrm{dk}\, i\left|\mu_{\mathrm{kg}}\right|^2 \Theta(\tau) e^{i(\omega_{\mathrm{kg}} - i\gamma_{\mathrm{kg}})\tau}\,, \tag{2}$$

where $\mu_{\mathrm{kg}} = \mu_{\mathrm{cg}}(\epsilon + q)/(\epsilon + i)$ denotes the $|g\rangle \rightarrow |k\rangle$ transition dipole moment with Fano parameter q and reduced energy $\epsilon = (\omega_{\mathrm{kg}} - \omega_{\mathrm{eg}})/\gamma_{\mathrm{e}}$. It is ω_{jg} the $|g\rangle \rightarrow |j\rangle$ transition frequencies, γ_{kg} the dephasing rate of the *k*-*g* coherence, $\gamma_{\mathrm{e}} \propto V^2$ the dissipation/tunneling rate from the $|e\rangle$ state into the continuum and $\Theta(\mathrm{t})$ the Heaviside step function. For simplicity we omitted the phase modulation term.

A Fourier transform yields the spectral signal

$$S(\omega) \propto \int dk\, i\left|\mu_{\mathrm{kg}}\right|^2 \frac{1}{\omega - \omega_{\mathrm{kg}} - i\gamma_{\mathrm{kg}}}\,. \tag{3}$$

For the dilute gas-phase sample probed in our study, we can assume $\gamma_{\mathrm{kg}} \ll \gamma_{\mathrm{e}}$. The real part of supplementary Eq. 3 is then

$$Re\{S(\epsilon, q)\} \propto \frac{(q + \epsilon)^2}{\epsilon^2 + 1}\,, \tag{4}$$

which describes the well-known Fano profile.

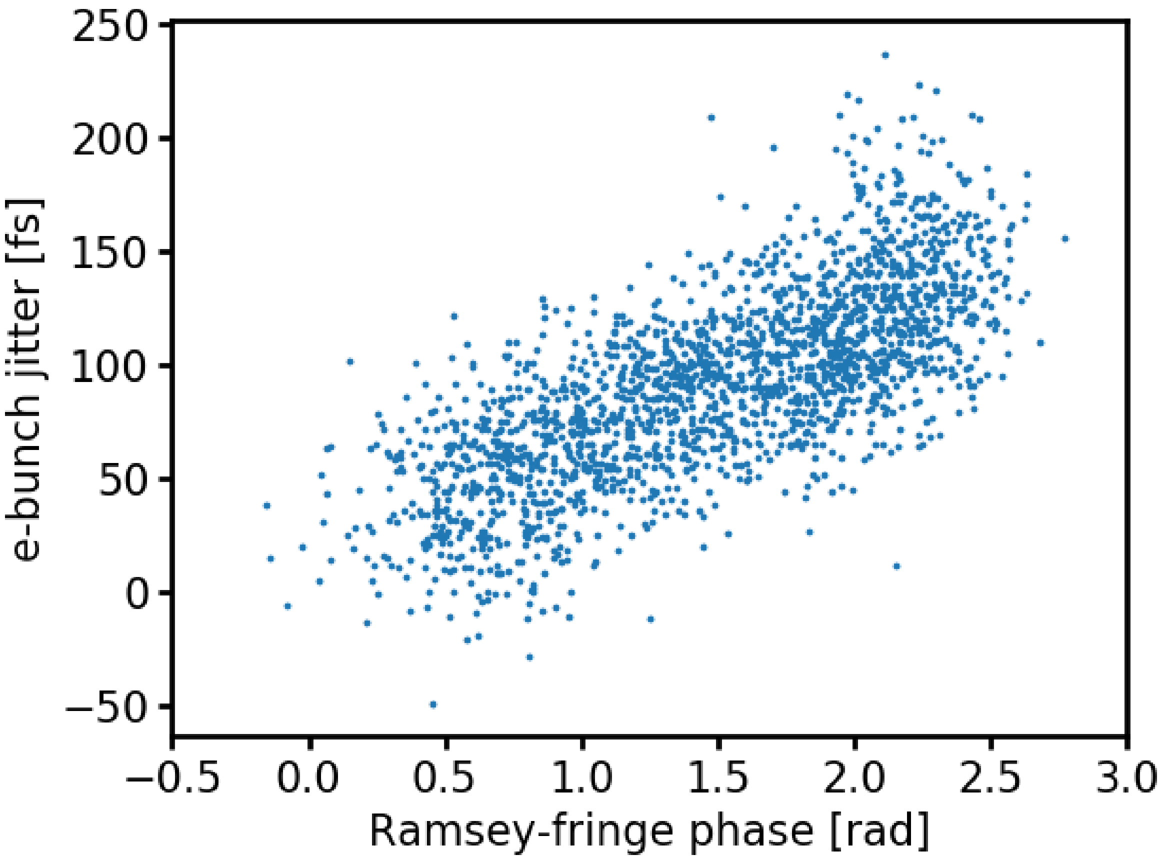

Supplementary Figure 1 Phase stability of the fifth harmonic for 2000 consecutive shots. A strong linear correlation, similar to the one in suppementary Ref. 2, is observed between the phase of the Ramsey-type fringe pattern on the beamline spectrometer and the timing jitter between seed pulses and electron-bunch. The electron bunch jitter has an RMS value of 42 fs and the phase jitter of 0.6 rad in this measurement.

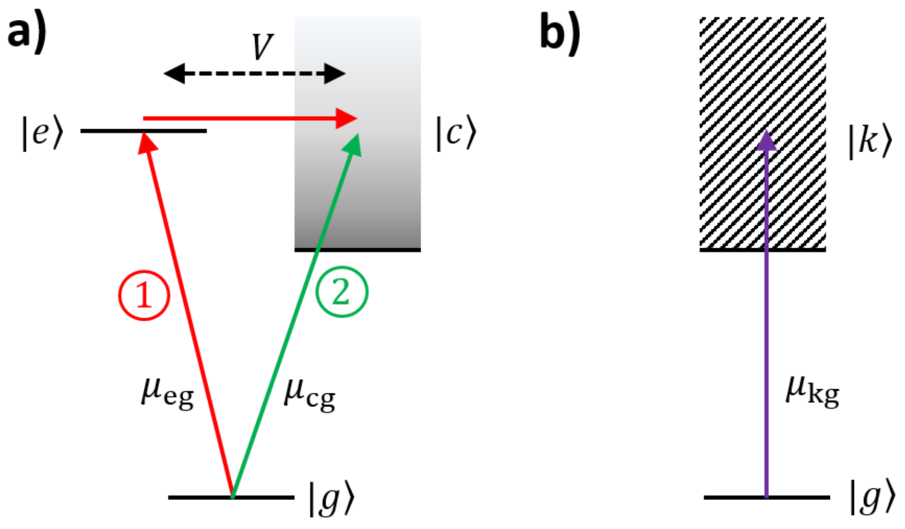

Supplementary Figure 2 Level diagram of a model Fano system in a) with $|g\rangle$ ground, $|e\rangle$ bound excited and $|c\rangle$ continuum states and $\mu_{\mathrm{eg}}, \mu_{\mathrm{cg}}$ corresponding transition dipole moments. V denotes the coupling between $|e\rangle$ and $|c\rangle$ states. b) shows the equivalent diagonalized system with $|g\rangle$ ground and $|k\rangle$ quasi-bound eigenstates of the Hamiltonian and μ_{kg} respective transition dipole moment.

Supplementary References:

1. Finkelstein-Shapiro, D., Poulsen, F., Pullerits, T. & Hansen, T. Coherent two-dimensional spectroscopy of a Fano model. *Phys. Rev. B* **94**, 205137 (2016).
2. Gauthier, D. et al. Generation of Phase-Locked Pulses from a Seeded Free-Electron Laser. *Phys. Rev. Lett*. **116,** 024801 (2016).
