# Supplementary material for: Tracking attosecond electronic coherences using phase-manipulated extreme ultraviolet pulses
Source: Nat Commun. 2020 Feb 14;11:883. doi: 10.1038/s41467-020-14721-2 (PMC7021897; doi:10.1038/s41467-020-14721-2)
Supplement: Supplementary file 2 — Peer Review File [file 41467_2020_14721_MOESM2_ESM.pdf]

Editorial Note: This manuscript has been previously reviewed at another journal that is not operating a transparent peer review scheme. This document only contains reviewer comments and rebuttal letters for versions considered at Nature Communications.

Reviewers' Comments:

Reviewer #1:
Remarks to the Author:
The authors have answered all the comments and questions, and revised the manuscript accordingly. The paper is now recommended for publication.

Reviewer #2:
Remarks to the Author:
The authors have addressed all my concerns and I can recommend publication in its present form.

REVIEWERS' COMMENTS:

Reviewer #1 (Remarks to the Author):

The authors have answered all the comments and questions, and revised the manuscript accordingly. The paper is now recommended for publication.

Reviewer #2 (Remarks to the Author):

The authors have addressed all my concerns and I can recommend publication in its present form.

Authors Response:

We thank the reviewers for their appreciation of our work and the recommendation for publication in Nature Communications.
